# Cardiovascular magnetic resonance imaging and clinical follow-up in patients with clinically suspected myocarditis after COVID-19 vaccination

**DOI:** 10.1016/j.jocmr.2024.101036

**Published:** 2024-03-11

**Authors:** Norain Talib, Matteo Fronza, Constantin Arndt Marschner, Paaladinesh Thavendiranathan, Gauri Rani Karur, Kate Hanneman

**Affiliations:** a Department of Medical Imaging, University Medical Imaging Toronto, University of Toronto, Toronto, Ontario, Canada; b Division of Cardiology, Peter Munk Cardiac Center, University Health Network, University of Toronto, Toronto, Ontario, Canada; c Toronto General Hospital Research Institute, University Health Network, University of Toronto, Toronto, Ontario, Canada

**Keywords:** CMR, Myocarditis, Vaccination, COVID-19

## Abstract

**Background:**

The purpose of this study was to evaluate cardiovascular magnetic resonance (CMR) findings and their relationship to longer-term clinical outcomes in patients with suspected myocarditis following coronavirus disease 2019 (COVID-19) vaccination.

**Methods:**

Consecutive adult patients who underwent clinically indicated CMR for evaluation of suspected myocarditis following messenger ribonucleic acid (mRNA)-based COVID-19 vaccination at a single center between 2021 and 2022 were retrospectively evaluated. Patients were classified based on the revised Lake Louise criteria for T1-based abnormalities (late gadolinium enhancement [LGE] or high T1 values) and T2-based abnormalities (regional T2-hyperintensity or high T2 values).

**Results:**

Eighty-nine patients were included (64% [57/89] male, mean age 34 ± 13 years, 38% [32/89] mRNA-1273, and 62% [52/89] BNT162b2). On baseline CMR, 42 (47%) had at least one abnormality; 25 (28%) met both T1- and T2-criteria; 17 (19%) met T1-criteria but not T2-criteria; and 47 (53%) did not meet either. The interval between vaccination and CMR was shorter in those who met T1- and T2-criteria (28 days, IQR 8–69) compared to those who met T1-criteria only (110 days, IQR 66–255, p < 0.001) and those who did not meet either (120 days, interquartile range (IQR) 80–252, p < 0.001). In the subset of 21 patients who met both T1- and T2-criteria at baseline and had follow-up CMR, myocardial edema had resolved and left ventricular ejection fraction had normalized in all at median imaging follow-up of 214 days (IQR 132–304). However, minimal LGE persisted in 10 (48%). At median clinical follow-up of 232 days (IQR 156–405, n = 60), there were no adverse cardiac events. However, mild cardiac symptoms persisted in 7 (12%).

**Conclusion:**

In a cohort of patients who underwent clinically indicated CMR for suspected myocarditis following COVID-19 vaccination, 47% had at least one abnormality at baseline CMR. Detection of myocardial edema was associated with the timing of CMR after vaccination. There were no adverse cardiac events. However, minimal LGE persisted in 48% at follow-up.

## Graphical abstract

**Figure:**
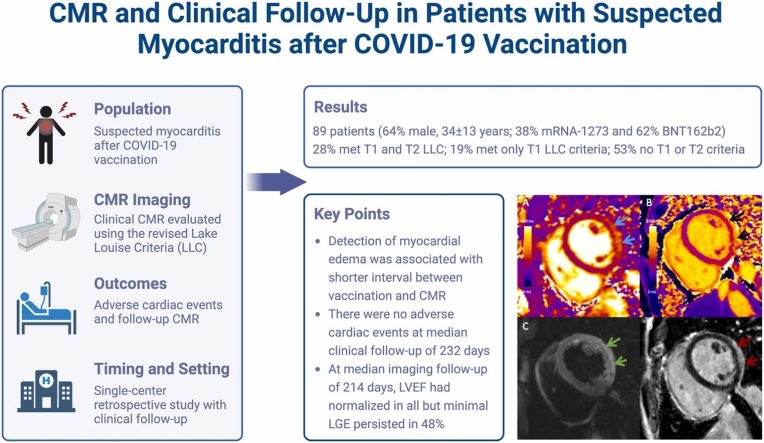

## 1 Introduction

Coronavirus disease 2019 (COVID-19) messenger ribonucleic acid (mRNA)-based vaccines including BNT152b2 (Pfizer-BioNTech, Mainz, Germany) and mRNA-1273 (Moderna, Cambridge, Massachusetts) are associated with elevated risk of myocarditis, particularly in younger men following the second dose [1]. Characteristic features of myocarditis are inflammation and myocyte damage. Cardiovascular magnetic resonance (CMR) imaging plays an important role in non-invasive evaluation of suspected acute myocarditis and is evaluated using revised Lake Louise Criteria (LLC) for acute myocardial inflammation [2], [3], [4]. Typical findings in acute myocarditis on CMR include subepicardial late gadolinium enhancement (LGE) and myocardial T2-hyperintensity at the basal to mid inferolateral wall. Elevated T2 signal reflects myocardial edema, which often resolves over weeks as inflammation subsides [5].

Prior studies have demonstrated that CMR abnormalities are typically less severe in myocarditis following COVID-19 mRNA vaccination compared to myocarditis following COVID-19 infection and other causes of myocarditis [6]. Data from small cohorts of patients suggest that most patients with myocarditis following COVID-19 vaccination have rapid resolution of symptoms with no adverse clinical outcomes at short- and mid-term clinical follow-up [7], [8]. However, a small study that utilized combined cardiac positron emission tomography magnetic resonance imaging (positron emission tomography-/magnetic resonance imaging (MRI)) demonstrated that 12% of participants with myocarditis following COVID-19 vaccination had persistent myocardial fluorodeoxyglucose (FDG)-uptake suggestive of inflammation at 2-month follow-up [9]. Currently, there is limited data on imaging abnormalities among broader cohorts of patients with suspected myocarditis following COVID-19 vaccination, including long-term clinical and imaging follow-up.

The purpose of this study was to evaluate CMR findings and their relationship to long-term clinical outcomes in patients with suspected myocarditis following COVID-19 vaccination.

## 2 Methods

### 2.1 Study design and participants

This retrospective cohort study was approved by the institutional ethics committee and the requirement for written informed consent was waived. Consecutive adult patients (≥18 years of age) with clinically suspected myocarditis who were referred to a tertiary hospital network for evaluation of myocarditis by clinical CMR between June 2021 and December 2022 were identified. Inclusion criteria were 1) new-onset cardiac symptoms (including chest pain, palpitations, or shortness of breath) within 14 days of mRNA-based COVID-19 vaccination, 2) fulfillment of clinical presentation criteria of the European Society of Cardiology diagnostic criteria for clinically suspected myocarditis [10], and 3) clinically indicated CMR. Exclusion criteria included CMR performed for follow-up of previously diagnosed myocarditis (if baseline imaging was not available for review) or research studies.

Clinical data on demographic characteristics, cardiac symptoms, vaccine administration history, and clinical outcomes were extracted from the electronic patient record. Adverse cardiac events, including death, arrhythmia (defined as sustained atrial or ventricular arrhythmia lasting at least 30 s), and heart failure hospitalization, were evaluated.

Baseline findings of 21 patients and short-term follow-up of 10 patients have been previously reported [6], [7]. The current study extends this analysis with a larger sample size, longer clinical follow-up, and inclusion of all patients referred for clinically indicated MRI for suspected myocarditis following COVID-19 vaccination at our center.

### 2.2 MRI technique

CMR studies were performed using 1.5T or 3T scanners (Magnetom AVANTOfit/SKYRAfit, Siemens Healthineers, Erlangen, Germany) with commercially available cardiac surface coils following current guidelines [11]. The MRI protocol included long-axis and a stack of short-axis balanced cine steady state-free precession (bSSFP) slices with complete ventricular coverage (slice thickness 8 mm and 2 mm inter-slice gap) and a stack of black-blood T2-weighted spectral attenuated inversion-recovery images at matching short-axis locations. A single mid-ventricular short-axis T1 and T2 mapping slice was acquired using a modified Look-Locker Inversion Recovery technique for native T1 mapping (5(3)3 inversion grouping) and a matching T2 map using a T2-prep technique with read-out varying with external field-strength (bSSFP at 1.5T and Fast Low Angle Shot at 3T). Pixel-based T1 and T2 maps were automatically generated on the scanner with application of inline motion correction algorithms. LGE images were acquired in short- and long-axis using a two-dimensional phase-sensitive inversion recovery technique starting 12 min after administration of intravenous contrast (0.15 mmol/kg body weight of gadobutrol, Bayer Healthcare, Berlin, Germany).

### 2.3 MRI analysis

CMR studies were analyzed independently by two experienced fellowship trained observers (N.T. and M.F.) who were blinded to all clinical information using commercially available tools (Circle cmr42; Circle Cardiovascular Imaging, Calgary, Alberta, Canada). Left and right ventricular volumes and function and left ventricular mass were measured using automated contour detection with manual correction if required as per established standards [12]. Presence of LGE and regional T2-weighted hyperintensity were evaluated visually (present or not) globally and according to the American Heart Association (AHA) 17-segment model [13]. For assessment of LGE, the predominant pattern was classified as subendocardial, mid-wall, subepicardial, or transmural. T1 and T2 mapping source images were visually evaluated for artifacts and any segments with artifact were excluded from analysis. Maximum T1 and T2 values were measured by manually drawing a region of interest in areas of visually maximum myocardial values based on the color map, with a minimum region of interest size of 0.5 cm^2^ [9], [14]. As per current guidelines, abnormal maximum T1 and T2 values were defined as 2 standard deviations (SD) above the mean of sequence specific local reference values (high T2 defined as >52 ms at 1.5T and >45 ms at 3T; high T1 defined as >1067 ms at 1.5T and >1289 ms at 3T) [15], [16]. To facilitate combined analysis of multi-scanner data, T1 and T2 values were converted to a *z*-score using scanner-specific local reference values (patient value − mean of reference range)/(SD of reference range) [17]. In this case, *z*-scores provide an assessment of how many SD each patient’s T1 or T2 value is above or below the mean for the normal range for each scanner. Patients were classified according to the revised LLC based on the presence or absence of T1 criteria (LGE or high T1 mapping values) and T2 criteria (regional T2-hyperintensity or high T2 mapping values) into one of three groups: 1) no T1 or T2 criteria met, 2) either T1 or T2 criteria met but not both, and 3) both T1 and T2 criteria met [1].

### 2.4 Statistical analysis

Categorical data are presented as counts (percentages) and continuous variables as means ± SDs and medians (IQRs). All continuous data were tested for normal distribution using the Shapiro-Wilk test. Comparisons between groups were conducted using one-way analysis of variance for continuous variables with normal distribution and Kruskal-Wallis test for continuous variables with non-normal distribution, with post hoc tests for significance using Bonferroni correction. Fisher exact test was used to compare categorical variables. Wilcoxon signed rank test was used to compare baseline and follow-up MRI parameters. All tests were two-tailed, and p values less than 0.05 were considered statistically significant. Analysis was performed using STATA software v14.1 (StataCorp, College Station, Texas), and data were visualized with GraphPad Prism 9.0.2 (GraphPad Software, Inc., La Jolla, California).

## 3 Results

### 3.1 Baseline characteristics

**Fig. 1:**
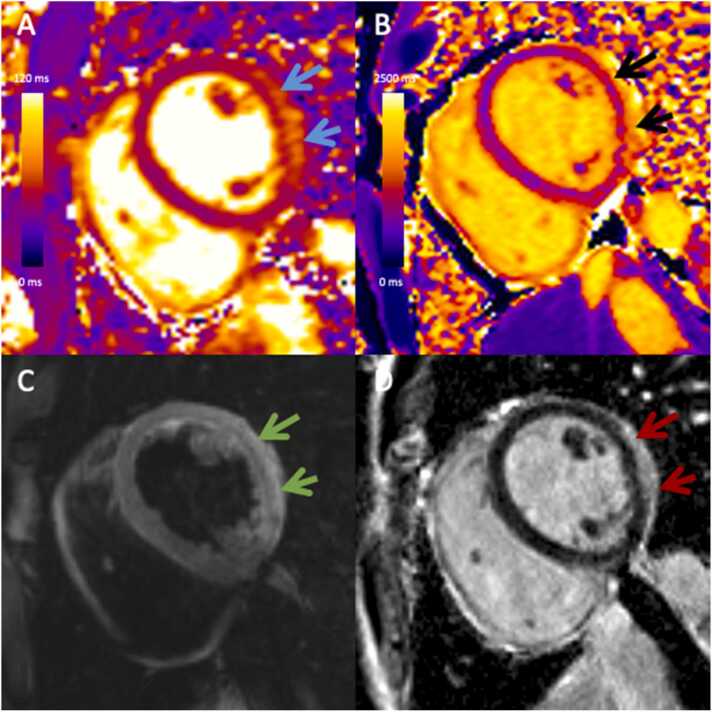
CMR in a 26-year-old male with myocarditis meeting both T1 and T2 Lake Louise Criteria after COVID-19 vaccination. He presented with chest pain and elevated troponin levels (peak at 10,351 ng/mL) after the second dose of mRNA-1273 vaccine. CMR performed 7 days post vaccination (1.5T) demonstrates (A) high T2 mapping values (62 ms, blue arrows), (B) high T1 mapping values (1303 ms, black arrows), (C) regional T2-hyperintensity (green arrows), and (D) late gadolinium enhancement (red arrows) at the subepicardial mid inferolateral and anterolateral wall. CMR, cardiovascular magnetic resonance

**Fig. 2:**
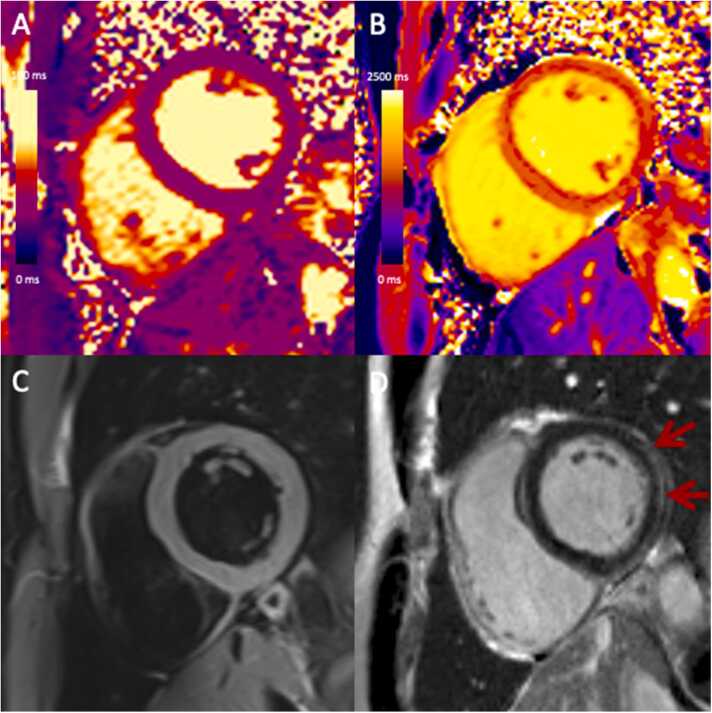
CMR in a 32-year-old male with suspected myocarditis after COVID-19 vaccination meeting T1 Lake Louise Criteria but not T2 criteria. He presented with chest pain after the second dose of mRNA-1273 vaccine. CMR performed 60 days after vaccination (3T) demonstrates (A) normal T2 mapping values (43 ms), (B) borderline T1 mapping values (1262 ms), (C) no regional T2-hyperintensity, and (D) mild late gadolinium enhancement (red arrows) at the subepicardial basal inferolateral wall.

Ninety-two patients were evaluated for eligibility and three were excluded due to lack of baseline imaging. Eighty-nine patients were included (64% male, 34 ± 13 years). Overall, 47% had at least one abnormality identified on baseline CMR; 25 (28%) met both T1 and T2 criteria, 17 (19%) met T1 criteria but not T2 criteria, and 47 (53%) did not meet either T1 or T2 criteria, Table 1 and Fig. 1, Fig. 2. None of the patients met only T2 but not T1 criteria. There were no differences in age (p = 0.10) or sex (p = 0.29) between groups.

**Table 1:** Patient characteristics.

	All patients (n = 89)	No T1 or T2 LLC (n = 47)	T1 but no T2 LCC (n = 17)	T1 and T2 LLC (n = 25)	p value^a^
Age, year	34 ± 13	36 ± 13	37 ± 14	30 ± 14	0.10
Male (%)	57 (64)	30 (64)	8 (47)	19 (76)	0.17
Height, cm	173 ± 9	171 ± 10	170 ± 8	176 ± 8	0.10
Weight, kg	76 ± 17	80 ± 19	70 ± 12	78 ± 16	0.24
BSA, m^2^	1.9 ± 0.2	1.9 ± 0.2	1.8 ± 0.2	1.9 ± 0.2	0.20
Vaccine type (BNT152b2)^b^	52 (62)	29 (69)	11 (65)	12 (48)	0.24
Vaccine type (mRNA-1273)^b^	32 (38)	13 (31)	6 (35)	13 (52)	0.24
Interval between vaccine and CMR, days (IQR)	92 (IQR 31–157)	120 (IQR 80–252)	110 (IQR 66–255)	28 (IQR 8–69)^c^ b	<0.001
Comorbidities^d^					
Diabetes (%)	0 (0)	0 (0)	0 (0)	0 (0)	>0.99
Hypertension (%)	4 (6)	1 (4)	3 (21)	0 (0)	0.03
Dyslipidemia (%)	2 (3)	1 (4)	1 (7)	0 (0)	0.07
Smoking (%)	6 (10)	4 (17)	1 (7)	1 (4)	0.42
Baseline arrhythmia (%)	1 (1)	1 (2)	0 (0)	0 (0)	>0.99
Hospital admission, No. (%)	23 (26)	5 (11)	2 (12)	16 (64)^c^	<0.001
Symptoms at presentation^e^					
Palpitations (%)	18 (28)	11 (42)	3 (21)	4 (17)	0.13
Chest pain (%)	53 (79)	17 (59)	12 (86)	24 (100)^c^	<0.001
Shortness of breath (%)	13 (20)	8 (30)	3 (21)	2 (8)	0.16

Variables are presented as mean ± standard deviation, median with interquartile range in parentheses, or number with percentage in parentheses.

*BSA,* body surface area; CMR, cardiovascular magnetic resonance; LLC, Lake Louise criteria, IQR, interquartile range.

a p-value for three-group comparison using one-way analysis of variance, Kruskal-Wallis, or Fisher exact test as appropriate for the type of data.

b Data on last vaccine type available in 84 patients (42 patients with no T1 or T2 LLC, 17 patients with T1 but no T2 LLC, and 25 with T1 and T2 LLC).

c Post hoc test for the difference vs no myocarditis group, p < 0.05.

d Data on comorbidities available in 62 patients (24 patients with no T1 or T2 LLC, 14 patients with T1 but no T2 LLC, and 24 with T1 and T2 LLC).

e Data on symptoms available in 67 patients (29 patients with no T1 or T2 LLC, 14 patients with T1 but no T2 LLC, and 24 with T1 and T2 LLC).

The last vaccine dose type was mRNA-1273 in 38% and BNT162b2 in 62%, and vaccine type did not differ between groups (p = 0.24). All patients included had new-onset cardiac symptoms following vaccination, including chest pain in 79%, shortness of breath in 20%, and palpitations in 28%. Overall, 23 patients (34%) required hospital admission in the acute phase. At baseline, one patient who did not meet either T1 or T2 criteria had documented paroxysmal atrial fibrillation. There were no other baseline arrhythmias.

The interval between last vaccine dose and CMR was shorter in the group who met both T1 and T2 criteria (28 days, IQR 8–69) compared to the group who met only T1 criteria (110 days, IQR 66–255, p < 0.001) and those who did not meet either T1 or T2 criteria (120 days, IQR 80–252, p < 0.001). However, the interval between those who met T1 criteria and those who did not meet either T1 or T2 criteria did not differ significantly (p > 0.99). Overall, 84% of patients who had baseline CMR performed within 30 days of symptom onset had both T1- and T2-based abnormalities on CMR.

### 3.2 CMR

CMR characteristics are provided in Table 2. LGE was present in 36 patients overall (41%), most frequently in a subepicardial pattern (64% in group who met T1 and T2 criteria and 59% in the group who met T1 criteria only). In the group that met both T1 and T2 criteria, T2 hyperintensity was present in 15 patients (65%) and high T2 mapping was present in 19 patients (76%). Left ventricular ejection fraction (LVEF) and presence of regional wall motion abnormalities did not differ between groups (p = 0.50 and p = 0.28, respectively).

**Table 2:** Baseline CMR findings.

	All patients (n = 88)	No T1 or T2 LLC (n = 47)	T1 but no T2 LCC (n = 17)	T1 and T2 LLC (n = 25)	p value^a^
Left ventricle					
LVEDVi, mL/m^2^	79 ± 16	77 ± 18	82 ± 14	79 ± 15	0.58
LVMi, g/m^2^	51 ± 11	50 ± 11	53 ± 14	52 ± 10	0.60
LVEF, %	57 ± 4.6	58 ± 4.5	58 ± 5.8	57 ± 3.8	0.50
LV regional wall motion abnormality (%)	5 (6%)	1 (2%)	2 (12%)	2 (8%)	0.28
Right ventricle					
RVEDVi, mL/m^2^	86 ± 20	85 ± 18	88 ± 17	85 ± 22	0.85
RVEF, %	53 ± 5.7	52 ± 5.1	52 ± 5.1	53 ± 5.2	0.95
RV regional wall motion abnormality (%)	0 (0%)	0 (0%)	0 (0%)	0 (0%)	>0.99
Left atrial area, cm^2^	20 ± 6.3	20 ± 4.0	20 ± 4.3	17 ± 5.2	0.96
Right atrial area, cm^2^	17 ± 5.2	17 ± 5.7	17 ± 3.9	18 ± 5.1	0.52
Tissue characterization					
LGE presence (%)^b^	36 (41)	0 (0)	15 (88)^c^	21 (84)^c^	<0.001
No LGE (%)^b^	51 (58)	45 (98)	2 (12)^c^	4 (16)^c^	<0.001
Subendocardial LGE (%)^b^	0 (0)	0 (0)	0 (0)	0 (0)	<0.001
Mid-wall linear LGE (%)^b^	3 (3)	0 (0)	2 (12)	1 (4)	<0.001
Mid-wall patchy LGE (%)^b^	6 (7)	0 (0)	2 (12)	4 (16)	<0.001
Subepicardial LGE (%)^b^	26 (30)	0 (0)	10 (59)^c^	16 (64)^c^	<0.001
Transmural LGE (%)^b^	0 (0)	0 (0)	0 (0)	0 (0)	<0.001
LGE, number of segments	0 (IQR 0–1)	0 (IQR 0–0)	1 (IQR 1–2)^c^	2 (IQR 1–3)^c^	<0.001
Hyperintense T2-weighted signal (%)^d^	15 (18)	0 (0)	0 (0)	15 (65)^c^	<0.001
High native T1 (%)^e^	19 (23)	0 (0)	3 (19)	16 (64)^c^	<0.001
Maximum native T1 value^e^	0.8 ± 3.9	0.5 ± 0.8	1.1 ± 1.2	1.1 ± 7.1	0.76
High native T2 (%)^f^	20 (24)	1 (2)	0 (0)	19 (76)^c^	<0.001
Maximum native T2 value^f^	0.8 ± 3.9	0.5 ± 0.8	1.1 ± 1.2	1.1 ± 7.1	0.76
Any T1 category (%)	42 (47)	0 (0)	17 (100)^c^	25 (100)^c^	<0.001
Any T2 category (%)	25 (28)	0 (0)	0 (0)	25 (100)^c^	<0.001
Pericardium					
Pericardial enhancement or edema (%)	18 (21)	1 (2)	5 (29)^c^	12 (50)^c^	<0.001

Variables are presented as median with interquartile range in parentheses or number with percentage in parentheses.

*LGE,* late gadolinium enhancement; *LV,* left ventricle; *LVEDVi*, left ventricular end-diastolic volume indexed to body surface area; *LVEF,* left ventricular ejection fraction; *LVMi*, indexed left ventricular mass; *RV,* right ventricle;, *RVEDVi*, right ventricular end-diastolic volume indexed to body surface area; *RVEF,* right ventricular ejection fraction, LLC, Lake Louise criteria; LGE, late gadolinium enhancement.

a p value for three-group comparison using one-way analysis of variance, Kruskal-Wallis, or Fisher exact test as appropriate for the type of data.

b Data on LGE presence and LGE patterns available in 86 patients (45 patients with no T1 or T2 LLC, 16 patients with T1 but no T2 LLC, and 25 with T1 and T2 LLC).

c Post hoc test for the difference vs no myocarditis group, p < 0.05.

d Data on hyperintense T2-weighted signal available in 85 patients (46 patients with no T1 or T2 LLC, 16 patients with T1 but no T2 LLC, and 23 with T1 and T2 LLC).

e Data on high native T1 and maximum native T1 available in 84 patients (43 patients with no T1 or T2 LLC, 16 patients with T1 but no T2 LLC, and 25 with T1 and T2 LLC).

f Data on high native T2 and maximum native T2 available in 85 patients (44 patients with no T1 or T2 LLC, 16 patients with T1 but no T2 LLC, and 25 with T1 and T2 LLC).

### 3.3 Follow-up CMR

**Fig. 3:**
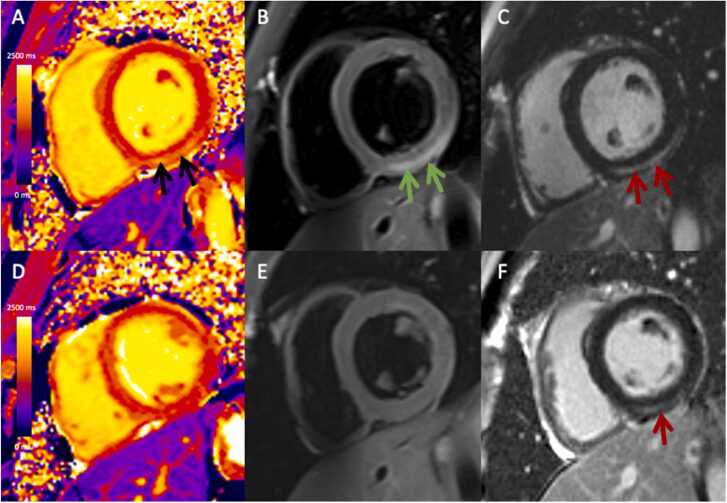
Baseline and follow-up CMR in a 21-year-old male with myocarditis after COVID-19 vaccination (BNT162b2). He presented with chest pain and baseline CMR was performed 8 days after vaccination (3T) demonstrating (A) high T1 mapping values (1404 ms, black arrows), (B) regional T2-hyperintensity (green arrows), and (C) late gadolinium enhancement (red arrows) at the subepicardial basal to mid inferior to inferolateral wall. Follow-up CMR was performed 142 days after vaccination (1.5T) demonstrating (D) normal T1 mapping values (1222 ms), (E) complete resolution of T2-hyperintensity, and (F) mild persistent late gadolinium enhancement at the subepicardial basal to mid inferior wall (red arrows) although decreased in extent compared to baseline.

Follow-up CMR was available in a subset of 21 patients who met both T1 and T2 criteria at baseline, at a median interval of 214 days (IQR 132–304) after the baseline MRI, Fig. 3. In this subset, myocardial edema had resolved in all at follow-up. LVEF increased (56 ± 3% to 58 ± 4%, p = 0.03) while native T1 and T2 decreased (median z-score 0.9 [IQR 0.2–1.8] vs 0.1 [IQR −0.5 to 0.5], p = 0.01 and 0.6 [IQR −0.3 to 1.9] vs 0.0 [IQR −0.7 to 0.7], p < 0.001, respectively). However, minimal LGE persisted in 10 patients at follow-up (48%).

### 3.4 Clinical follow-up

Clinical follow-up was available in 60 patients (67%) with median clinical follow-up duration of 232 days [IQR 156–405] (24/47 in the no T1 or T2 criteria group, 12/17 in the T1 criteria only group, and 24/25 in the T1 and T2 criteria group based on baseline CMR). There were no adverse cardiac events (death, sustained atrial or ventricular arrhythmia lasting ≥30 s, or heart failure hospitalization). Overall, 53 patients (88%) had complete resolution of symptoms and seven (12%) reported mild persistent cardiac symptoms at follow-up (three [13%] in the baseline no T1 or T2 criteria group, two [17%] in the baseline T1 criteria only group, and two [8%] in the baseline T1 and T2 criteria group). Persistent symptoms at follow-up were not associated with any of the baseline clinical or CMR parameters.

## 4 Discussion

In this retrospective cohort study of 89 patients referred for clinical CMR for suspected myocarditis following mRNA-based COVID-19 vaccination, 47% had at least one abnormality identified on baseline CMR. Overall, 28% had both T1- and T2-based abnormalities, 19% had T1-based abnormalities but no T2-based abnormalities, and 53% did not have either T1- or T2-abnormalities on baseline CMR. None of the patients met T2-based criteria but not T1-based criteria. Interval resolution of edema on follow-up CMR highlights the importance of early baseline imaging for detection of edema in the setting of suspected myocarditis. Absence of adverse cardiac events at median 232-day clinical follow-up is reassuring. However, 12% of patients had mild persistent symptoms and 48% had persistent LGE. Strengths of this study compared to prior reports include a cross-sectional analysis of baseline findings among a broad cohort of symptomatic patients referred for clinical CMR for evaluation of suspected myocarditis following COVID-19 vaccination, along with long-term clinical and imaging follow-up.

Consistent with prior CMR studies, the most common pattern of abnormalities on CMR among patients meeting LLC for myocarditis was subepicardial LGE, often involving the basal to mid inferolateral wall [6], [18]. Myocardial edema, detected by myocardial T2 hyperintensity or high T2 mapping values, was present in 28% of patients at baseline and was associated with the timing of CMR after vaccination. Among the subset of patients with CMR follow-up, myocardial edema had resolved in all on repeat CMR, highlighting the importance of early CMR after symptoms onset for detection of edema. Of note, lack of a T2-based abnormality on CMR does not necessarily mean that a patient did not have prior acute myocarditis. Many patients in the group with T1 abnormalities only at baseline, had LGE in a pattern typical for myocarditis and met clinical criteria for myocarditis. In this group, myocardial edema may have resolved by the time of the CMR. The revised LLC criteria highlight that in the appropriate clinical context, one T1-based or T2-based criteria could be consistent with acute inflammation [1]. Earlier imaging with documentation of myocardial edema is helpful to confirm the diagnosis with higher specificity and to establish the acuity of the findings.

Follow-up CMR demonstrated normalization of left ventricular function and interval decrease in LGE along with resolution of myocardial edema. These findings are consistent with the typical rapid decrease in myocardial inflammation in other causes of myocarditis over days to weeks [5]. However, minimal LGE without edema was present in 48% of patients at follow-up. In the setting of myocarditis, persistent LGE without corresponding edema at follow-up likely reflects replacement fibrosis [4]. In a prior study, short-term CMR follow-up at median 100 days post-vaccination in a cohort of 13 patients demonstrated residual LGE in 62% [7]. Similarly, follow-up CMR at 5–6 months in nine patients with post-vaccine myocarditis demonstrated persistent LGE in 78% [19]. The lower proportion of persistent LGE in the current study could be related to long-term follow-up at median 214 days post-vaccination.

The presence of LGE is associated with adverse cardiac events in patients with non-vaccine acute myocarditis [20]. However, the significance of LGE is uncertain in patients post-myocarditis with recovered normal left ventricular systolic function. Myocarditis following COVID-19 vaccination may be associated with a more favorable prognosis compared with other causes of myocarditis [21]. In a retrospective cohort study of 866 patients, patients with myocarditis following COVID-19 mRNA vaccination had lower incidence of adverse outcomes (including mortality and heart failure) compared to those with post-viral myocarditis [21]. Our analysis confirms low adverse events rates in patients with myocarditis following COVID-19 vaccination at median 232 days post-vaccination. However, long-term follow-up studies are needed to evaluate the clinical significance of altered myocardial T1 values and LGE in patients with recovered left ventricular function after COVID-19 vaccine myocarditis.

Recent studies have also investigated cardiac imaging in patients not meeting clinical criteria for myocarditis. A recent prospective study evaluated 67 patients with CMR ≤14 days before and ≤14 days after COVID-19 vaccination with no detectable change in native T1, T2, extra-cellular volume, LVEF, or LGE between pre- and post-vaccine MRI [22]. New symptoms within 14 days of vaccination included chest pain in 22% and shortness of breath in 16%; however, none of the participants met LLC for myocarditis on MRI. Similarly, a recent prospective study found no evidence of myocardial inflammation on combined FDG-positron emission tomography/MRI 2 months after COVID-19 vaccination in symptomatic and asymptomatic participants not meeting criteria for myocarditis [9]. These findings are reassuring that COVID-19 vaccination does not typically cause subclinical myocardial injury despite presence of new-onset cardiac symptoms in a minority of patients.

## 5 Limitations

Our study has limitations including a modest sample size. More than one MRI scanner was used for imaging. To address this, we interpreted parametric mapping values in the context of scanner specific local reference ranges and calculated *z*-scores for T1 and T2 values. The timing of CMR after symptom onset varied. However, these reflect a real-world clinical cohort where there is often variability in the timing of imaging based on the patient’s clinical presentation and local CMR wait times. We did not adjust for differences in timing of imaging and did not restrict our analysis to patients with CMR within a specific time range as we were interested in evaluating timing of imaging in relation to CMR findings in this population. Only mid-ventricular T1 and T2 mapping slices were examined, which could underestimate maximum T1 and T2 values if regional disease was only present in other areas of myocardium. However, LGE and T2-weighted imaging were performed with coverage of the entire myocardium. Imaging and clinical follow-up were only available in a subset of patients, reflecting a real-world clinical cohort with variable follow-up. However, the number of patients with follow-up is larger than prior studies and demographics did not differ in these sub-groups compared to the entire cohort. Finally, histologic confirmation of myocarditis was not available as endomyocardial biopsy is not frequently performed at our center for this reason [23].

## 6 Conclusion

In a cohort of 89 patients who underwent clinically indicated CMR for suspected myocarditis following COVID-19 vaccination, 47% had at least one abnormality at baseline. Detection of myocardial edema was associated with the timing of MRI after vaccination. There were no adverse cardiac events. However, minimal LGE persisted in 48%, warranting ongoing follow-up.

## Funding

None.

## Author contributions

**Gauri Rani Karur:** Formal analysis, Methodology, Writing – review and editing. **Paaladinesh Thavendiranathan:** Conceptualization, Formal analysis, Writing – review and editing. **Constantin Arndt Marschner:** Data curation, Methodology, Writing – review and editing. **Matteo Fronza:** Conceptualization, Data curation, Writing – review and editing. **Norain Talib:** Data curation, Writing – original draft. **Kate Hanneman:** Conceptualization, Data curation, Formal analysis, Methodology, Supervision, Writing – original draft, Writing – review and editing.

## Ethics approval and consent

This retrospective study was approved by the institutional (University Health Network) research ethics board. The requirement for written informed consent was waived.

## Consent for publication

Obtained.

## Declaration of competing interests

The authors declare that they have no known competing financial interests or personal relationships that could have appeared to influence the work reported in this paper.
